# P-711. Persistence of Immunogenicity and Safety of the Respiratory Syncytial Virus Prefusion F Protein Vaccine (RSVPreF3 OA) Administered to Adults 50–59 Years of Age (YOA)

**DOI:** 10.1093/ofid/ofae631.907

**Published:** 2025-01-29

**Authors:** Murdo Ferguson, Tino F Schwarz, Sebastián A Núñez, Juan Rodríguez García, Marek Mital, Carlos Zala, Bonavuth Pek, Bernhard Schmitt, Nicole Toursarkissian, Dolores Ochoa Mazarro, Josef Grosskopf, Christine Voors-Pette, Hemalini Mehta, Hiwot Amare Hailemariam, Caroline Portaels, Magali de Heusch, Bruno Salaun, Silvia Damaso, Marie-Pierre David, Dominique Descamps, Judith Hill, Corinne Vandermeulen, Veronica Hulstrøm

**Affiliations:** Colchester Research Group, Truro, Canada, Truro, Nova Scotia, Canada; Klinikum Würzburg Mitte, Campus Juliusspital, Würzburg, Germany, Wuerzburg, Bayern, Germany; Centro Medico Maffei, Buenos Aires, Argentina, Buenos Aires, Buenos Aires, Argentina; Son Espases University Hospital, Palma de Mallorca, Mallorca, Balearic Islands, Spain, Mallorca, Islas Baleares, Spain; Clinical Agnieszka Mital Centrum Badan Clinic, Elblag, Poland, Warsaw, Mazowieckie, Poland; Vacunar, Sede Las Cañitas, Buenos Aires, Argentina, Buenos Aires, Buenos Aires, Argentina; Clinique de Pneumologie et du Sommeil de Lanaudière, Saint-Charles-Borromée, Québec, Canada, Saint-Charles-Borromée, Quebec, Canada; Studienzentrum Mainz Mitte, Mainz, Germany, Mainz, Rheinland-Pfalz, Germany; Praxis Dr. Med. Nicole Toursarkissian, Berlin, Germany, Berlin, Berlin, Germany; Hospital Universitario de La Princesa, Instituto de Investigación Sanitaria La Princesa (IP), Universidad Autónoma de Madrid (UAM), Madrid, Spain, Madrid, Madrid, Spain; Praxis Dr. Med. Josef Großkopf, Wallerfing, Germany, Wallerfing, Bayern, Germany; QPS Netherlands B.V., Groningen, The Netherlands, Groningen, Groningen, Netherlands; Clinical Research Institute, Minneapolis, MN, USA, Minneapolis, Minnesota; GSK, Wavre, Belgium, Wavre, Brabant Wallon, Belgium; GSK, Wavre, Belgium, Wavre, Brabant Wallon, Belgium; GSK, Wavre, Brabant Wallon, Belgium; GSK, Wavre, Brabant Wallon, Belgium; GSK, Wavre, Belgium, Wavre, Brabant Wallon, Belgium; GSK, Wavre, Belgium, Wavre, Brabant Wallon, Belgium; GSK, Wavre, Belgium, Wavre, Brabant Wallon, Belgium; GSK, Wavre, Belgium, Wavre, Brabant Wallon, Belgium; GSK, Wavre, Belgium, Wavre, Brabant Wallon, Belgium; GSK, Wavre, Belgium, Wavre, Brabant Wallon, Belgium

## Abstract

**Background:**

The immune response induced by RSVPreF3 OA in adults 50–59 YOA, including adults with chronic conditions that increase the risk for RSV disease, was non-inferior in terms of RSV-A/-B neutralization titers compared to that in older adults (≥ 60 YOA) at 1 month (M) post-vaccination.

**Figure:**
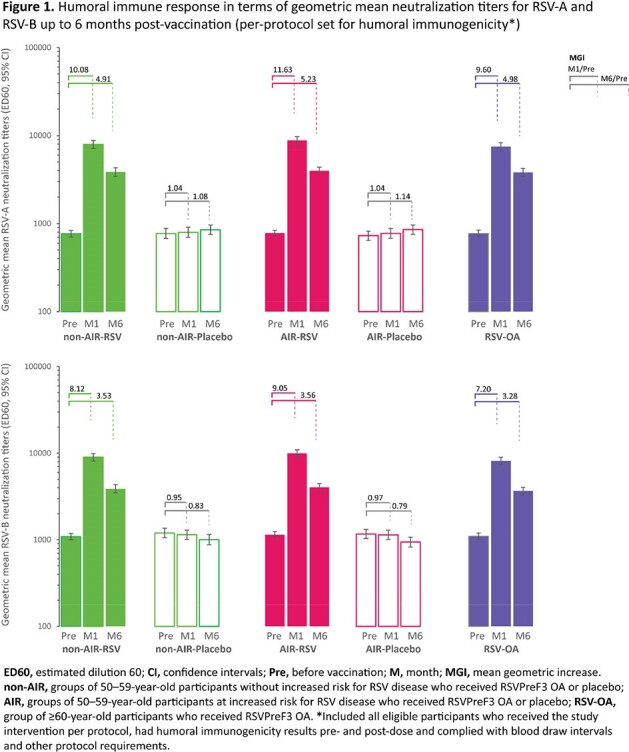

**Methods:**

This was a phase 3, observer-blind, placebo-controlled multi-country study (NCT05590403). Adults 50–59 YOA at increased risk (AIR) for RSV disease due to specific chronic conditions and not at increased risk for RSV disease (non-AIR) 50–59 YOA adults were randomized (2:1) to receive 1 dose of RSVPreF3 OA (AIR-RSV, non-AIR-RSV) or placebo (AIR-placebo, non-AIR-placebo). A control group of adults ≥ 60 YOA received RSVPreF3 OA (RSV-OA). We assessed the immune response at 1M and 6M post-vaccination. Humoral immunity (HI) and cell-mediated immunity (CMI) were assessed in terms of RSV-A/-B neutralization titers and RSVPreF3-specific CD4+ T-cell frequencies. Safety up to study end (12M post-vaccination) was also assessed.

**Figure:**
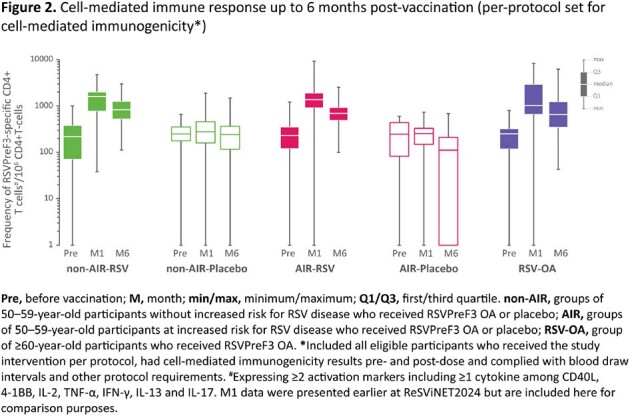

**Results:**

1533 participants received RSVPreF3 OA or placebo. In all RSV groups, RSV-A/-B neutralization titers (Figure 1) and RSVPreF3-specific CD4+ T-cell frequencies (Figure 2) increased substantially from pre-vaccination to 1M post-vaccination and remained above baseline levels at 6M (4.9–5.2-fold for RSV-A and 3.3–3.6-fold RSV-B neutralization titers). Within 6M post-vaccination, 3.9% (AIR-RSV), 0.8% (non-AIR-RSV), 2.1% (AIR-placebo, non-AIR-placebo) and 2.4% (RSV-OA) of participants reported serious adverse events (SAEs). Eight potential immune-mediated diseases (AIR-RSV: 4; AIR-placebo: 1; RSV-OA: 3) were reported within 6M post-vaccination. Of these, one case of cold type hemolytic anemia (RSV-OA, onset at day 53 post-vaccination, also SAE) was considered vaccine-related by the investigator. Five deaths (AIR-RSV: 4; AIR-placebo: 1), none considered vaccine-related, were reported up to study end (12M).

**Conclusion:**

At 6M post-vaccination, HI and CMI remained above baseline both in RSVPreF3 OA-vaccinated adults 50–59 YOA with/without chronic conditions and in adults ≥60 YOA (RSV-OA). Up to 12M, the safety profile in adults 50–59 YOA was similar to the safety profile in those ≥ 60 YOA and was considered acceptable.

**Funding**: GSK

**Disclosures:**

**Murdo Ferguson, MD**, GSK: Study related payments for training and the conduct of the study as a study site **Tino F. Schwarz, Prof. Dr. MD**, AstraZeneca: Honoraria|Bavarian Nordic: Advisor/Consultant|Bavarian Nordic: Honoraria|Biogen: Honoraria|Biontech: Advisor/Consultant|Biontech: Honoraria|CSL Vifor: Honoraria|CSL-Seqirus: Advisor/Consultant|CSL-Seqirus: Honoraria|Diasorin: Honoraria|GSK: Advisor/Consultant|GSK: Honoraria|Janssen-Cilag: Honoraria|Merck-Serono: Honoraria|Moderna: Advisor/Consultant|Moderna: Honoraria|MSD: Honoraria|Novavax: Advisor/Consultant|Novavax: Honoraria|Pfiser: Honoraria|Roche: Honoraria|Sanofi-Aventis: Honoraria|Synlab: Honoraria|Takeda: Advisor/Consultant|Takeda: Honoraria **Sebastián A. Núñez, Dr.**, GSK: Support for attending meetings and/or travel **Juan Rodríguez García, MD**, GSK: Advisor/Consultant|GSK: Grant/Research Support|GSK: Honoraria|Pfizer: Honoraria|Pfizer: Support for attending meetings and/or travel|Sanofi: Honoraria|Sanofi: Support for attending meetings and/or travel **Carlos Zala, MD**, GSK: Grant/Research Support|GSK: Support for attending meetings and/or travel (EACS 2023) **Josef Grosskopf, MD**, GSK: Advisor/Consultant|GSK: Expert Testimony|GSK: Grant/Research Support|GSK: Honoraria|GSK: Support for attending meetings and/or travel|Lilly: Grant/Research Support|New Amsterdam Pharma: Grant/Research Support|Novartis: Grant/Research Support|Pharmalog: Grant/Research Support|Syneos: Grant/Research Support|Winecker Pharma: Grant/Research Support **Christine Voors-Pette, MD**, QPS Netherlands B.V.: Ex-Employee of QPS Netherlands B.V. **Hiwot Amare Hailemariam, MD, PhD**, GSK: Salary as GSK employee with stock options|GSK: Stocks/Bonds (Public Company) **Caroline Portaels, DVM**, GSK: Salary as GSK employee with stock options|GSK: Stocks/Bonds (Public Company) **Magali de Heusch, PhD**, GSK: Salary as GSK employee with stock options|GSK: Stocks/Bonds (Public Company) **Bruno Salaun, PhD**, GSK: Salary as GSK employee with stock options|GSK: Stocks/Bonds (Public Company) **Silvia Damaso, MSc**, GSK: Salary as GSK employee with stock options|GSK: Stocks/Bonds (Public Company) **Marie-Pierre David, Master in Statistics**, GSK: As GSK employee, I’m part of a patent application|GSK: Salary as GSK employee with stock options|GSK: Stocks/Bonds (Public Company) **Dominique Descamps, MD**, GSK: Salary as GSK employee with stock options|GSK: Stocks/Bonds (Public Company) **Judith Hill, Dr.**, GSK: Salary as GSK employee with stock options|GSK: Stocks/Bonds (Public Company) **Corinne Vandermeulen, MD, PhD**, GSK: Salary as GSK employee with stock options|GSK: Stocks/Bonds (Public Company) **Veronica Hulstrøm, MD, PhD**, GSK: Salary as GSK employee with stock options|GSK: Stocks/Bonds (Public Company)

